# Coenzyme Q_4_ is a functional substitute for coenzyme Q_10_ and can be targeted to the mitochondria

**DOI:** 10.1016/j.jbc.2024.107269

**Published:** 2024-04-06

**Authors:** Laura H. Steenberge, Sean Rogers, Andrew Y. Sung, Jing Fan, David J. Pagliarini

**Affiliations:** 1 Department of Biochemistry, University of Wisconsin-Madison, Madison, Wisconsin, USA; 2 University of Wisconsin Medical Scientist Training Program, University of Wisconsin School of Medicine and Public Health, Madison, Wisconsin, USA; 3 Morgridge Institute for Research, Madison, Wisconsin, USA; 4 Department of Cell Biology and Physiology, Washington University School of Medicine, St Louis, Missouri, USA; 5 Department of Biochemistry and Molecular Biophysics, Washington University School of Medicine, St Louis, Missouri, USA; 6 Department of Genetics, Washington University School of Medicine, St Louis, Missouri, USA; 7 Department of Biomolecular Chemistry, University of Wisconsin School of Medicine and Public Health, Madison, Wisconsin, USA; 8 Department of Nutritional Sciences, University of Wisconsin-Madison, Madison, Wisconsin, USA

**Keywords:** coenzyme Q10 (CoQ10), ubiquinone, mitochondrial respiratory chain complex, mitochondrial therapeutics, membrane lipid, bioenergetics, antioxidant, ferroptosis, pyrimidine biosynthesis

## Abstract

Coenzyme Q10 (CoQ_10_) is an important cofactor and antioxidant for numerous cellular processes, and its deficiency has been linked to human disorders including mitochondrial disease, heart failure, Parkinson’s disease, and hypertension. Unfortunately, treatment with exogenous CoQ_10_ is often ineffective, likely due to its extreme hydrophobicity and high molecular weight. Here, we show that less hydrophobic CoQ species with shorter isoprenoid tails can serve as viable substitutes for CoQ_10_ in human cells. We demonstrate that CoQ_4_ can perform multiple functions of CoQ_10_ in CoQ-deficient cells at markedly lower treatment concentrations, motivating further investigation of CoQ_4_ as a supplement for CoQ_10_ deficiencies. In addition, we describe the synthesis and evaluation of an initial set of compounds designed to target CoQ_4_ selectively to mitochondria using triphenylphosphonium. Our results indicate that select versions of these compounds can successfully be delivered to mitochondria in a cell model and be cleaved to produce CoQ_4_, laying the groundwork for further development.

Coenzyme Q (CoQ) is a ubiquitous redox-active lipid with critical functions throughout the cell. It is composed of a polyisoprenoid tail that anchors it in lipid bilayers and a benzoquinone head group that can accept and donate electrons. CoQ is required for shuttling electrons from complexes I and II to complex III in the electron transport chain (ETC) and participates in myriad mitochondrial and extramitochondrial processes including pyrimidine biosynthesis, ferroptosis suppression, fatty acid oxidation, sulfide detoxification, and membrane antioxidation (1). These diverse and important functions are reflected in the wide spectrum of clinical diseases that arise from deficiency of CoQ_10_ (the predominant CoQ species found in humans). Genetic defects in CoQ_10_ biosynthesis cause phenotypes varying from nephropathy and myopathy to fatal multiorgan disease (2). In addition, secondary deficits in CoQ_10_ levels have been observed in aging (3) and in numerous common conditions such as neurodegenerative diseases (4), cardiomyopathy (5), and primary mitochondrial diseases (6).

CoQ_10_ supplementation has emerged as an attractive therapeutic candidate for these disorders. CoQ_10_ treatment has been the subject of numerous clinical trials and is among the most common supplements taken in the Western world (5). Despite widespread interest and use, CoQ_10_ treatment is frequently ineffective (7, 8, 9). Poor bioavailability and cell delivery due to the large size and extreme hydrophobicity of CoQ_10_ are often cited as reasons for treatment failure (1, 8, 10). Transportation of the lipophilic CoQ_10_ across the gut epithelium and through the aqueous environment of the body is a major barrier to effective supplementation, with only 2% of enterally administered CoQ reaching the bloodstream (11) and an even smaller fraction reaching mitochondria and other target membranes in the cell (12, 13). Thus, a less hydrophobic CoQ analog or an analog better directed to mitochondria could more efficiently reach target membranes and represent attractive alternatives for CoQ_10_ supplementation.

The hydrophobicity of CoQ is primarily determined by its long polyisoprenoid tail (Table 1), which plays a critical role in anchoring CoQ within the core of the lipid bilayer to facilitate its interactions with membrane-embedded proteins. The specific number of isoprene units in the tail (denoted by a subscript) varies between organisms. For example, humans, *Saccharomyces cerevisiae*, and *Escherichia coli* have ten, six, and eight isoprene units in the tail of their dominant CoQ form, respectively. Although the reason for this variation remains poorly understood, it suggests that reducing the hydrophobicity of the CoQ tail may not compromise its function in humans.

**Table 1:** Octanol-water partition coefficients (*P*) of CoQ species with increasing isoprenoid tail lengths as calculated using Advanced Chemistry Development (ACD) Software

Coenzyme Q species	Octanol-water partition coefficient (Log*P*)
Coenzyme Q_0_	0.12
Coenzyme Q_1_	2.61
Coenzyme Q_2_	4.65
Coenzyme Q_3_	6.68
Coenzyme Q_4_	8.72
Coenzyme Q_5_	10.75
Coenzyme Q_6_	12.79
Coenzyme Q_7_	14.82
Coenzyme Q_8_	16.86
Coenzyme Q_9_	18.89
Coenzyme Q_10_	20.93

CoQ analogs with tail modifications have been investigated as CoQ_10_ substitutes. For instance, idebenone, a CoQ mimetic approved for treatment of Leber hereditary optic neuropathy (14), consists of the benzoquinone head group of CoQ with a fully saturated 10-carbon acyl chain capped by a hydroxyl group in place of the isoprenoid tail. The dramatic decrease in hydrophobicity and structural differences likely prevent its direct substitution for CoQ_10_ in the ETC; rather, its cellular effects occur through entirely separate mechanisms (15, 16, 17). Moreover, studies involving CoQ species with shortened isoprenoid tail lengths have been met with inconsistent outcomes (18). These short-chain quinones can act as cellular antioxidants and donate cytoplasmic electrons to complex III in the context of complex I impairment (16, 18); however, their ability to directly substitute for CoQ_10_ has not been fully established. In yeast, CoQ_2_ can rescue CoQ-deficient respiratory growth (19); in *Drosophila*, CoQ_4_ can rescue impaired neural growth from loss of CoQ (20). In human systems, CoQ_6_ was unable to increase CoQ-dependent oxidative phosphorylation (OxPhos) activities in HL-60 cells (21), and CoQ_2_ did not increase ATP levels in CoQ-deficient fibroblasts (17). Conversely, a separate study showed that CoQ_4_ restored ATP levels and CoQ-dependent OxPhos in CoQ-deficient patient fibroblasts (22). In addition to potential benefits, shorter chain quinones have also been associated with adverse effects in cells, such as the induction of reactive oxygen species (ROS) and apoptosis (21, 23, 24, 25).

Mitochondria-targeting CoQ species have also been developed as potential CoQ_10_ substitutes. Most notably, the molecule MitoQ is composed of the CoQ head group linked to triphenylphosphonium (TPP), a mitochondriotropic moiety. While MitoQ rapidly accumulates in mitochondria and exhibits potent antioxidant behavior, the attached TPP prevents the head group from properly interacting with ETC complexes (26), and thus it is unable to substitute for CoQ_10_ in supporting OxPhos. Here, toward establishing a more effective CoQ therapeutic, we examined a series of CoQ tail lengths across multiple assays to clarify the minimum hydrophobicity required for CoQ to function in human cells and developed a series of CoQ analogs with reversible TPP linkages to explore their ability to enhance the functional delivery of CoQ.

## Results

### Short-chain CoQ analogs support OxPhos

**Figure 1:**
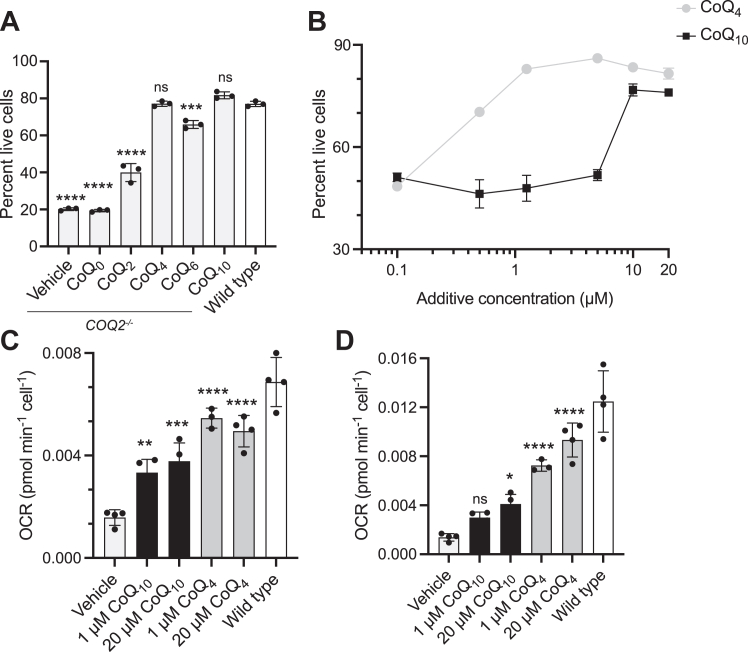
**Short-chain CoQ analogs support OxPhos.***A*, percent live wild-type or *COQ2*^*−/−*^ HepG2 cells after 72 h of incubation in galactose media with 20 μM indicated CoQ additive. Cells were pretreated with CoQ additives for 48 h to ensure sufficient uptake. Live cells defined as not staining for AAD-7 or annexin-V. *n* = three independent technical replicates, and error bars indicate standard deviation. Significance calculated using a one-way ANOVA. *B*, survival of *COQ2*^*−/−*^ HepG2 cells in galactose as in (*A*) over a titration of CoQ_4_ and CoQ_10_ concentrations. *n* = two independent technical replicates, and error bars indicate standard deviation. *C* and *D*, oxygen consumption rates (OCR) indicating (*C*) basal respiration and (*D*) maximal respiration after 3 μM FCCP treatment in *COQ2*^*−/−*^ and wild-type HepG2 cells after 24 h incubation with CoQ additives. *n* = four independent technical replicates, and error bars indicate standard deviation. Significance calculated using a one-way ANOVA. ns, not significant, ∗*p* < 0.05, ∗∗*p* < 0.01, ∗∗∗*p* < 0.001, ∗∗∗∗*p* < 0.0001. For exact *p* values, see Table S1. CoQ, coenzyme Q; OxPhos, oxidative phosphorylation.

Although decreasing the tail length of CoQ may improve its bioavailability, its tail must still be sufficiently lipophilic to partition CoQ into the inner leaflet of the mitochondrial inner membrane to interact with ETC complexes. To find this balance between aqueous solubility and functionality, we sought to identify the minimum isoprenoid tail length required for CoQ to support OxPhos. Compromised OxPhos function causes cell death in media containing galactose as the sole carbon source (27). Accordingly, HepG2 *COQ2*^*−/−*^ cells, which lack a key CoQ_10_ biosynthetic enzyme and are CoQ_10_ deficient, had increased rates of cell death in galactose media, which were reduced with 20 μM CoQ_10_ supplementation (Fig. 1*A*). Surprisingly, CoQ_4_ supplementation at this concentration was equally effective at preventing cell death. CoQ_2_ supplementation initially maintained viability in galactose (Fig. S1*A*), but extended treatment caused cell death, likely due to inherent toxicity of the compound (24, 25) (Fig. S1*B*). CoQ_4_ showed no evidence of cellular toxicity or increased ROS production at concentrations up to 100 μM (Fig. S2).

We repeated this analysis across a range of concentrations using CoQ_4_ and CoQ_10_ and found that, comparatively, 10-fold lower concentrations of the former were needed to rescue cell viability (Fig. 1*B*). Importantly, we confirmed that CoQ_4_ supplementation also increased basal and maximal respiration in CoQ_10_-deficient cells to a greater extent than CoQ_10_ supplementation (Fig. 1, *C* and *D*). CoQ_4_ supplementation additionally rescued mitochondrial polarization, ATP production, and oxygen consumption rate (OCR) in CoQ_10_-deficient cells grown in galactose media to a similar or greater extent than CoQ_10_ supplementation (Fig. S3). Overall, these results indicate that a tail length of four isoprene units is sufficient to support proper OxPhos function in human cells. Interestingly, while 1 μM of CoQ_10_ was not sufficient to rescue viability in galactose, it was able to increase basal OCR to a similar level as 20 μM CoQ_10_, suggesting that other functions of CoQ might contribute to viability in galactose media.

### CoQ_4_ can support ferroptosis defense and pyrimidine biosynthesis

**Figure 2:**
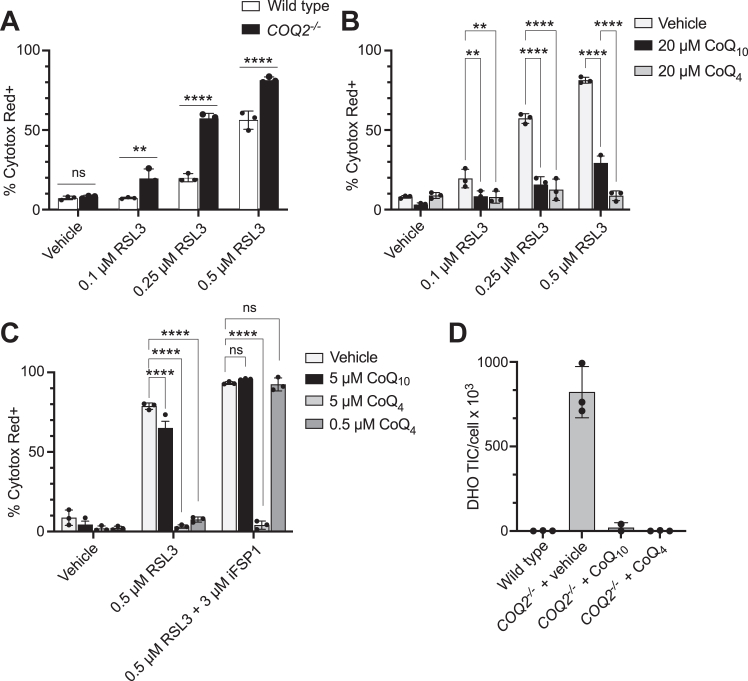
**CoQ**_**4**_**can participate in ferroptosis suppression and pyrimidine biosynthesis.***A*, cell death of wild-type and *COQ2*^*−/−*^ HepG2 cells after 24 h of treatment with GPX4-inhibitor RSL3. Cell death determined by high levels of Cytotox Red labeling. *n* = three independent technical replicates, and error bars indicate standard deviation. Significance calculated using a two-way ANOVA. *B*, cell death of *COQ2*^*−/−*^ HepG2 cells after 24 h of treatment with RSL3 following 24 h of preincubation with 20 μM CoQ_4_ or CoQ_10_. *n* = three independent technical replicates, error bars indicate standard deviation. Significance is calculated using a two-way ANOVA. *C*, cell death of *COQ2*^*−/−*^ HepG2 cells after 24 h of treatment with RSL3 or iFSP1 following 24 h of preincubation with indicated concentrations of CoQ_4_ or CoQ_10_. *n* = three independent technical replicates, and error bars indicate standard deviation. Significance calculated using a two-way ANOVA. *D*, total ion counts of dihydroorotate normalized to cell count in HAP1 wild-type and *COQ2*^*−/−*^ cells after 24 h of treatment with 20 μM CoQ_4_ or CoQ_10_. *n* = three independent technical replicates, and error bars indicate standard deviation. ns, not significant, ∗∗*p* < 0.01, ∗∗∗∗*p* < 0.0001. For exact *p* values see Table S1. CoQ, coenzyme Q; DHO,dihydroorotate.

Next, we examined whether CoQ_4_ could participate in CoQ_10_ roles beyond the ETC, such as ferroptosis defense and *de novo* pyrimidine biosynthesis. Reduced CoQ_10_ (CoQ_10_H_2_) at the plasma membrane helps prevent ferroptosis through radical trapping and suppression of lipid peroxidation and is regenerated by the oxidoreductase FSP1 for continued ferroptosis suppression (28, 29). This pathway works in parallel with another major ferroptosis defense mechanism mediated by GPX4, a hydroperoxidase that neutralizes lipid peroxides using glutathione. Thus, the HepG2 *COQ2*^*−/−*^ cells, which lack CoQ_10_ at the plasma membrane, were more sensitive to the GPX4 inhibitor RSL3 (Fig. 2*A*). This RSL3 sensitivity was decreased by CoQ_10_ supplementation and completely ablated by CoQ_4_ (Fig. 2*B*). Inhibition of FSP1 by its inhibitor iFSP1 blocked the ability of both CoQ_10_ and CoQ_4_ (at low concentrations) to prevent ferroptosis (Fig. 2*C*). Higher CoQ_4_ supplementation concentrations caused resistance to FSP1 inhibition, likely because there were sufficient amounts of reduced CoQ_4_ at the plasma membrane to preclude the need for FSP1-mediated regeneration.

CoQ also plays a key role in the pyrimidine *de novo* biosynthetic pathway as a cofactor for dihydroorotate dehydrogenase (DHODH); thus, HAP1 *COQ2*^*−/−*^ cells accumulated dihydroorotate (DHO), the substrate of DHODH (Fig. 2*D*). Supplementation of both CoQ_4_ and CoQ_10_ restored DHO to wildtype levels (Fig. 2*D*), suggesting that DHODH can use CoQ_4_ to synthesize orotate. Altogether, these results indicate that CoQ_4_ can fulfill roles of CoQ_10_ outside of the ETC.

### CoQ_4_ can ameliorate loss of cell viability due to statin treatment

**Figure 3:**
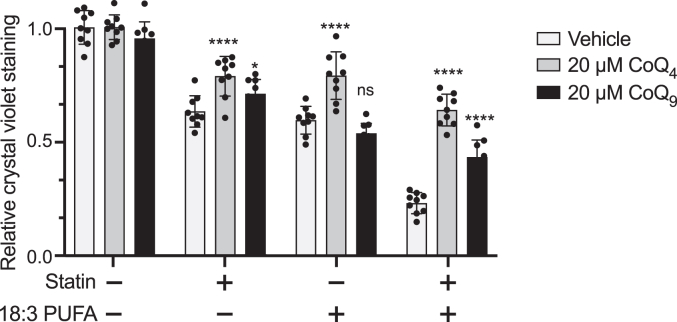
**CoQ**_**4**_**can ameliorate statin-induced loss of cell viability.** Cell viability of C2C12 myocytes after 48 h of treatment with 50 μM simvastatin, 50 μM linolenic acid (C18:3), or a combination. Cells were pretreated with 20 μM CoQ_4_ or CoQ_9_ for 24 h prior to addition of statin and polyunsaturated fatty acids to ensure adequate uptake. Cell viability was assessed with crystal violet, with normalization to vehicle-treated cells with no CoQ pretreatment. *n* = nine independent technical replicates, error bars indicate standard deviation. Significance calculated using a two-way ANOVA. ns, not significant, ∗∗∗∗*p* < 0.0001. For exact *p* values, see Table S1. CoQ, coenzyme Q.

Statin treatment can reduce levels of CoQ_10_ in the body (8), as the mevalonate pathway synthesizes the precursors for the isoprenoid tail of CoQ. This decrease in CoQ_10_ is hypothesized to contribute to myopathy associated with statin use in patients (30). Accordingly, CoQ_10_ treatment can decrease statin-induced toxicity (31) and mitochondrial dysfunction (32) in cellular and rodent models. To test the ability of CoQ_4_ to alleviate statin-induced toxicity, we measured cell viability of C2C12 cells treated with simvastatin in the presence or absence of the polyunsaturated fatty acid linolenic acid (C18:3) (used to further sensitize the cells to statin-induced loss of cellular CoQ). In each case, CoQ_4_ restored viability equally or more effectively than CoQ_9_ (the predominant form of CoQ in rodent cells) (Fig. 3). These results suggest a potential therapeutic application for CoQ_4_ in alleviating statin-related dysfunction in clinical settings.

### CoQ_4_ can be preferentially enriched in mitochondria

While CoQ_4_ is much less hydrophobic than CoQ_10_, it still contains 20 aliphatic carbons in its tail and retains considerable lipophilicity (Table 1). Therefore, there will likely still be significant barriers to its bioavailability and cellular delivery. We hypothesized that a reversible linkage to TPP, a mitochondria-targeting group, could increase the delivery of functional CoQ_4_. TPP is a well-studied lipophilic cation known to selectively transport cargo into mitochondria, driving concentrations of cargo in the mitochondrial matrix 100- to 1000-fold higher than extracellular concentrations. Moreover, TPP-conjugated compounds often bypass traditional transport mechanisms, instead directly passing through membranes to reach the mitochondrial matrix (33). Exogenous CoQ taken up by the cell primarily becomes trapped in lysosomes during transport (12, 13); thus, bypassing the cellular transport mechanisms with TPP could increase the amount of CoQ reaching target membranes.

**Figure 4:**
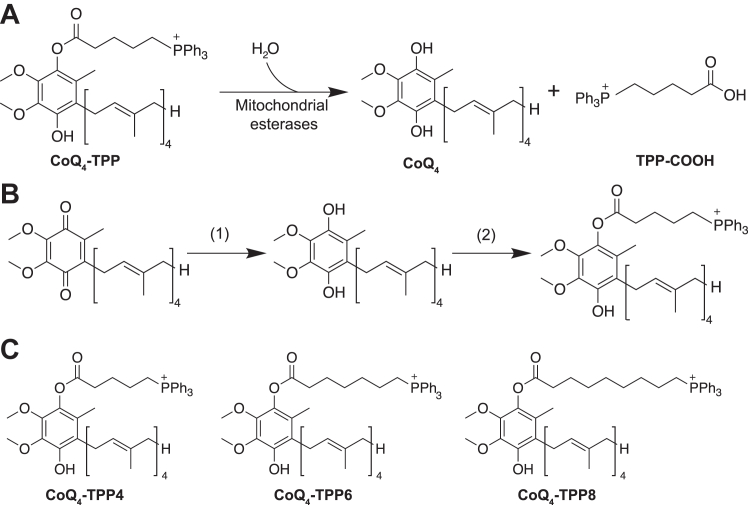
**Schematic of CoQ**_**4**_**-TPP hydrolysis and synthetic scheme.***A*, schematic of CoQ_4_-TPP hydrolysis by mitochondrial esterases to produce CoQ_4_ and a carboxy-TPP side product (TPP-COOH). *B*, chemical synthesis of CoQ_4_-TPP. Reaction conditions: (1) NaBH_4_ in methanol/isopropanol (2) 4-carboxybutyl triphenylphosphonium bromide, N-(3-dimethylaminopropyl)-N'-ethylcarbodiimide, 4-dimethylaminopyridine in dichloromethane. *C*, structures of CoQ_4_-TPP compounds synthesized for this study. CoQ, coenzyme Q; TPP, triphenylphosphonium.

**Figure 5:**
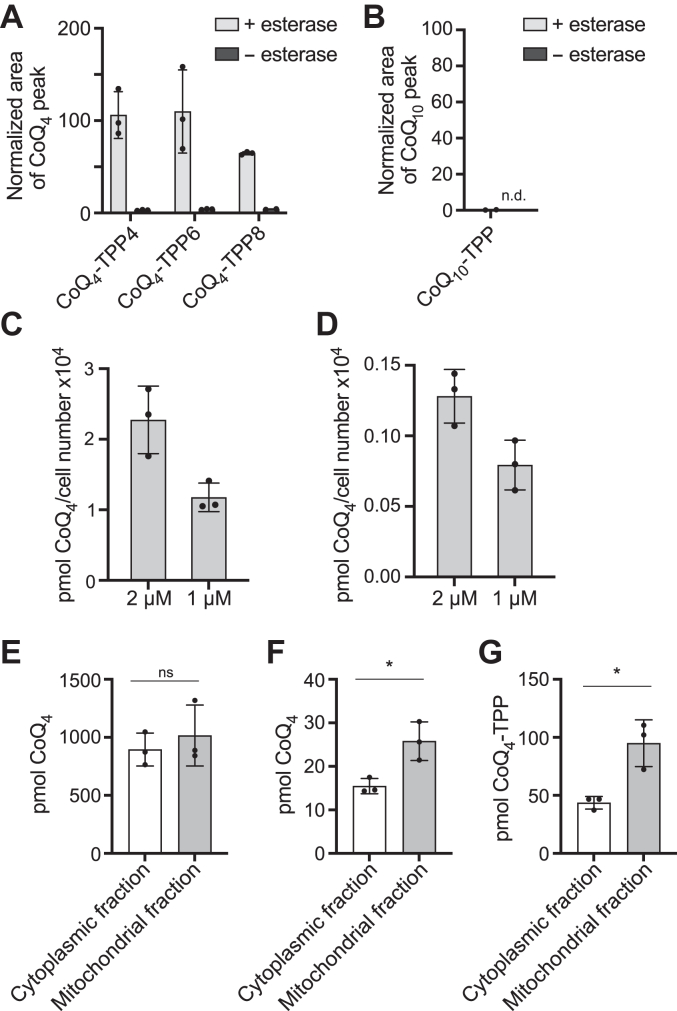
**CoQ**_**4**_**-TPP can be hydrolyzed to produce CoQ**_**4**_**.***A* and *B*, CoQ production from CoQ-TPP species after 24 h of incubation with porcine liver esterase, normalized to equivalent CoQ_4_ (*A*) or CoQ_10_ (*B*) area indicating 100% turnover. CoQ levels measured by HPLC-ECD. *n* = three independent technical replicates, and error bars indicate standard deviation. *C* and *D*, levels of CoQ_4_ after 24 h of incubation of wild-type HepG2 cells with unmodified CoQ_4_ (*C*) or CoQ_4_-TPP (*D*) measured by HPLC-ECD after cellular lipid extraction. *n* = three independent technical replicates, and error bars indicate standard deviation. *E* and *F*, CoQ_4_ levels in different subcellular compartments after 3 h of incubation of HepG2 cells with 10 μM CoQ_4_ (*E*) or CoQ_4_-TPP4 (*F*). *G*, CoQ_4_-TPP levels in different subcellular compartments after 3 h of incubation of HepG2 cells with 10 μM CoQ_4_-TPP4. *n* = three independent technical replicates, and error bars indicate standard deviation. Significance calculated with a two-tailed, unpaired *t* test. ns, not significant, ∗*p* < 0.05. For exact *p* values see Table S1. CoQ, coenzyme Q; TPP, triphenylphosphonium.

Studies with MitoQ have demonstrated that the irreversible attachment of TPP to CoQ prevents proper interactions with complex I and complex III, likely due to steric hindrance and improper membrane portioning. Therefore, we attached the TPP moiety to the head group of CoQ through an ester linkage (Fig. 4*A*). Esters are often labile in cells and can be hydrolyzed by resident nonspecific esterases to remove TPP-containing moieties (34, 35). We synthesized three CoQ_4_-TPP compounds with varying acyl linker chain lengths (Fig. 4*B*). Porcine liver esterase, a representative esterase, hydrolyzed all three compounds to a large extent *in vitro* (Fig. 5*A*), while a similar CoQ_10_-TPP compound was unable to be hydrolyzed (Fig. 5*B*). Incubating cells with CoQ_4_-TPP resulted in the dose-dependent production of free CoQ_4_ (Fig. 5*D*), although the resulting levels were 10-fold lower than levels from treatment with exogenous unmodified CoQ_4_ (Fig. 5*C*). Importantly, while unmodified CoQ_4_ treatment resulted in comparable amounts of CoQ_4_ in the cytoplasmic and mitochondrial fractions of the cell, CoQ_4_-TPP increased relative CoQ_4_ levels in the mitochondrial fraction compared to the cytoplasmic fraction (Figs. 5, *E*–*G* and S4), despite having diminished cellular uptake compared to CoQ_4_. Unfortunately, further investigations of CoQ_4_-TPP were limited by the toxicity of the compound (Fig. S5). While optimization of the delivery system to reduce toxicity and improve hydrolysis is required, these data provide proof-of-concept that CoQ_4_ can be reversibly linked to a mitochondria-targeting group and preferentially delivered to mitochondria, providing a platform for further study into CoQ targeting strategies.

## Discussion

Stemming from its myriad critical cellular roles, CoQ_10_ deficiency leads to a variety of human diseases. CoQ_10_ supplementation is often recommended for numerous conditions including primary mitochondrial diseases, heart failure, Parkinson’s disease, and hypertension (5, 36). CoQ_10_ is widely used as a nutritional supplement, with a global market size estimated to be nearly 600 million USD per year (8). Unfortunately, while CoQ_10_ supplementation has clear benefits in isolated cases (5, 37, 38), evidence supporting its clinical efficacy is often weak, and treatment failure is common, likely because the extreme hydrophobicity and large size of CoQ_10_ limits its uptake and distribution to target membranes. Here, we further demonstrate that the less hydrophobic CoQ_4_ can fulfill multiple functions of CoQ_10_ in a cellular model, positing that it could be a viable therapeutic alternative for CoQ supplementation, and show that CoQ_4_ can be preferentially targeted to mitochondria through linkage to TPP.

Surprisingly, we found that CoQ_4_ could rescue CoQ-deficiency phenotypes in cells at much lower treatment concentrations than CoQ_10_. This likely reflects increased CoQ_4_ delivery to target membranes rather than enhanced CoQ_4_ functionality. Indeed, in a reconstituted system, complex I displayed decreased catalytic efficacy with shorter CoQ species including CoQ_4_ (39). CoQ_4_ must reach multiple cellular membranes to fulfill CoQ’s various cellular roles. Its movement throughout the cell could be mediated by the endomembrane system, as has been shown for CoQ_10_ (40), or could occur through a separate mechanism. Given that CoQ_4_ has been shown to exchange between phospholipid bilayers of two vesicle populations while CoQ_10_ cannot (19), it is possible that CoQ_4_ does not require the same level of active transport between membranes as CoQ_10_ but instead can move through the cell without relying on dedicated protein machinery.

Assessing the bioavailability and cellular delivery of CoQ_4_ in an animal model will provide further insight into its potential as a CoQ_10_ substitute. While CoQ_4_ is much less hydrophobic than CoQ_10_, it still possesses a highly hydrophobic 20-carbon tail and could experience similar bioavailability barriers as CoQ_10_. Idebenone, a CoQ analog with a fully saturated 10-carbon tail, is much less lipophilic than CoQ_10_ yet is still poorly water soluble, which presents many challenges to its use (41, 42, 43). Thus, additional strategies to boost CoQ_4_ delivery will likely be required to develop an effective therapeutic.

Toward further improving CoQ delivery, we trialed reversibly attaching TPP to the CoQ head group, as TPP can drive cargo molecules to high concentrations intracellularly and specifically in mitochondria. Encouragingly, we observed release of free CoQ_4_ that was enriched in mitochondria. Unexpectedly, there was a substantial amount of CoQ_4_ and CoQ_4_-TPP in the cytoplasmic fraction as well. We suspect that this discrepancy from the usual profound enrichment of TPP compounds in mitochondria is due to the known disruption of the inner mitochondrial membrane potential caused by hydrophobic TPP molecules (44, 45, 46), which would decrease the electrochemical gradient driving CoQ_4_-TPP into mitochondria and likely also contribute to the observed toxicity. A less disruptive mitochondria targeting group, such as the novel TPP-CF_3_ derivatives (45), could potentially target CoQ_4_ more effectively. Alternative targeting groups could additionally alter the lipophilicity of the molecule and could result in improved cellular uptake compared to the CoQ_4_-TPP. Additionally, we surmise that the toxicity of our compound arises from the unhydrolyzed CoQ_4_-TPP, as joint treatment with unmodified CoQ_4_ and TPP-COOH resulted in high intracellular levels without evident toxicity. Therefore, more labile linker constructs would likely decrease the toxicity of the compound while also leading to higher levels of CoQ_4_ enrichment. Overall, our work encourages further exploration of less hydrophobic CoQ analogs in therapeutic regimens and provides proof-of-concept that CoQ species can be reversibly linked to a targeting moiety to enhance delivery to mitochondria.

## Experimental procedures

### General cell culture

HepG2 lines (ATCC) were cultured in Dulbecco's Modified Eagle Medium (DMEM) (Thermo) supplemented with 10% heat-inactivated fetal bovine serum (FBS) (R&D Systems, S11550) and 1x penicillin-streptomycin (Pen/Strep; Thermo, 15140122) at 37 ˚C and 5% CO_2_. The Genome Engineering and iPSC Center at Washington University in St Louis generated the HepG2 *COQ2*^*−/−*^ cell lines. HAP1 lines (Horizon Discovery) were cultured in Iscove’s Modified Dulbecco’s Medium supplemented with 10% heat-inactivated FBS and 1x penicillin–streptomycin at 37 ˚C and 5% CO_2_. C2C12 lines (ATCC) were cultured in DMEM (Thermo) supplemented with 10% heat-inactivated FBS and 1x penicillin–streptomycin at 37 ⁰C and 5% CO_2_. Lines were regularly tested for mycoplasma contamination. Cell counts were obtained using a Muse Cell Analyzer (Luminex) and the Muse Count and Viability Assay (Luminex).

### Galactose rescue

Wildtype and *COQ2*^−/−^ HepG2 cells were plated at 75,000 cells/well in 6-well plates. After allowing cells to adhere overnight, medium was exchanged to DMEM containing either vehicle (isopropanol), CoQ_0_ (Sigma, D9150), CoQ_2_ (Sigma, C8081), CoQ_4_ (Sigma, C2470), CoQ_6_ (Avanti, 900150O), or CoQ_10_ (Sigma, C9538) in isopropanol. After 48 h of incubation, cells were washed with Dulbecco’s phosphate buffered saline (dPBS), and medium was replaced with glucose-free DMEM containing 10 mM of galactose, 1 mM pyruvate, and 50 μg/ml uridine along with CoQ additives or vehicle. After 72 h, cell apoptosis was assessed by the Muse Annexin-V and Dead Cell Kit (Luminex). For shorter CoQ_2_ supplementation, cells were incubated with 20 μM CoQ_4_ for 24 h prior to galactose media replacement, and apoptosis was assessed after 48 h in galactose.

### Seahorse measurements

An Agilent Seahorse XFe96 Analyzer was used to determine OCRs. HepG2 wildtype and *COQ*2^*−/−*^ cells were plated at 30K cells per well in XF96 microplates (Agilent, 102416-100) with media containing CoQ species. The next day, cell media were exchanged for XF assay medium supplemented with 25 mM glucose, 1 mM pyruvate, 4 mM glutamine (Agilent, 03680-100), and CoQ species (Sigma). Oxygen consumption rate was measured at baseline and after injections of 2 μM oligomycin, 3 μM carbonyl cyanide 4-(trifluoromethoxy)phenylhydrazone (FCCP), and 0.5 μM rotenone/antimycin A (Agilent, 103015-100). For galactose OCR measurements, HepG2 wildtype and *COQ*2^*−/−*^ cells were preincubated with either vehicle, 20 μM CoQ_4_ (Sigma, C2470), or 20 μM CoQ_10_ (Sigma, C9538) in isopropanol for 72 h, then plated at 20K cells/well with CoQ species and allowed to adhere overnight. Cells were washed with dPBS, and medium was replaced with glucose-free DMEM containing 10 mM of galactose, 1 mM pyruvate, and 50 μg/ml uridine. The next day, cell media were exchanged for XF assay medium supplemented with 25 mM glucose, 1 mM pyruvate, and 4 mM glutamine (Agilent, 03680-100), and baseline oxygen consumption rates were measured. For all measurements, rates were normalized to cell count per well obtained immediately prior to XF assay medium exchange, obtained by Sartorius IncuCyte S3 live-cells analysis system.

### Ferroptosis sensitivity

Wildtype and *COQ2*^−/−^ HepG2 cells were plated at 25,000 cells/well in a 24-well plate and allowed to adhere overnight. Medium was exchanged to DMEM with 1 mM pyruvate and 50 μg/ml uridine along with either vehicle (isopropanol) or CoQ additives (Sigma) in isopropanol. After 48 h of incubation, cells were washed with dPBS. Media were replaced with DMEM with 1 mM pyruvate, 50 μg/ml uridine, and IncuCyte Cytotox Red Reagent (Fisher, NC1015259); along with RSL3 (Sigma, SML2234), iFSP1 (Fisher, NC1755669), or vehicle (DMSO). Cell death was quantified after 24 h using a Sartorius IncuCyte S3 live-cells analysis system.

### Dihydroorotate and ATP measurements

For DHO measurements, HAP1 wildtype and *COQ*2^*−/−*^ cells were plated at 500,000 cells/well in a 6-well plate and allowed to adhere overnight. Media were exchanged to DMEM containing either vehicle, 20 μM CoQ_4_ (Sigma, C2470) or 20 μM CoQ_10_ (Sigma, C9538) in isopropanol. After 24 h of incubation, cells were washed three times with cold dPBS, then incubated at −80 ˚C with cold LC-MS grade 80:20 methanol/water (v/v) for 15 min. Cells collected and centrifuged at 16,000*g* for 5 min. Supernatant was collected and dried under nitrogen flow. For ATP measurements, HepG2 wildtype and *COQ*2^*−/−*^ cells were preincubated with either vehicle, 20 μM CoQ_4_ (Sigma, C2470) or 20 μM CoQ_10_ (Sigma, C9538) in isopropanol for 72 h, then plated at 500,000 cells/well in a 6-well plate with CoQ species and allowed to adhere overnight. Cells were washed with dPBS, and medium was replaced with glucose-free DMEM containing 10 mM of galactose, 1 mM pyruvate, and 50 μg/ml uridine. After 24 h, metabolites were extracted as above. To measure the metabolite species, samples were resuspended in LC-MS grade water. Samples were analyzed using a Thermo Q-Exactive mass spectrometer coupled to a Vanquish Horizon UHPLC. Analytes were separated on a 100 × 2.1 mm, 1.7 μM Acquity UPLC BEH C18 Column (Waters), with a 0.2 ml min^−1^ flow rate and with a gradient of solvent A (97:3 H_2_O/methanol, 10 mM TBA, 9 mM acetate, pH 8.2) and solvent B (100% methanol). The gradient is as follows: 0 min, 5% B; 2.5 min, 5% B; 17 min, 95% B; 21 min, 95% B; and 21.5 min, 5% B. Data were collected in full-scan negative mode. Setting for the ion source was as follows: 10 aux gas flow rate, 35 sheath gas flow rate, 2 sweep gas flow rate, 3.2 kV spray voltage, 320 °C capillary temperature, and 300 °C heater temperature. The metabolites reported here were identified based on exact m/z and retention times determined with chemical standards.

### Mitochondrial membrane potential analysis

HepG2 wildtype and *COQ2*^*−/−*^ cells were plated at 4,000 cells/well in a 24-well plate and allowed to adhere overnight. Media were exchanged to glucose-free DMEM containing 10 mM of galactose, 1 mM pyruvate, and 50 μg/ml uridine with either vehicle, 5 μM CoQ_4_ (Sigma, C2470), or 5 μM CoQ_10_ (Sigma, C9538) in isopropanol. After 48 h of incubation, cells were washed with dPBS and incubated for 5 min with vehicle or 6.66 μM carbonyl cyanide 4-(trifluoromethoxy)phenylhydrazone (FCCP), then 100 nM TMRM was added. After 30 min, red fluorescence and cell number were determined using a Sartorius IncuCyte S3 live-cells analysis system.

### Statin toxicity assay

C2C12 cells were plated at 10,000 cells/well in a 96-well plate and allowed to adhere overnight. The next day, medium was replaced with fresh DMEM containing either vehicle (isopropanol) or 20 μM CoQ additive (Sigma) in isopropanol. After 24 h, cells were washed with dPBS, and media were replaced with DMEM containing 10 mM galactose with varying concentrations of simvastatin (Sigma, S6196) in isopropanol and/or linolenic acid (Sigma, L2376) in isopropanol. After 48 h, media were removed, and cells were washed three times with dPBS. Crystal violet staining solution (0.5% crystal violet in 25% methanol) was added to the cells and allowed to incubate for 20 min with gentle rocking. After removing the staining solution, cells were washed three times with dPBS, and remaining crystal violet was dissolved with 100% methanol. Absorbance was read at 540 nm in a microplate reader (Cytation3).

### ROS measurements and cell growth assay

Cells were plated at 40,000 cells/well in a 24-well plate and allowed to adhere overnight. Cell media were exchanged to DMEM containing 5 μM dichlorodihydrofluorescein diacetate (Abcam, ab113851) and incubated for 30 min at 37 °C. Cells were washed twice with dPBS, and medium was replaced with DMEM containing the CoQ additives (Sigma), menadione (Sigma, M5625), or vehicle (isopropanol). Using a Sartorius IncuCyte S3 live-cells analysis system, green fluorescence was determined after 24 hours of incubation to obtain ROS measurements, and cell number was obtained after 48 hours of incubation to calculate fold change.

### Synthesis of CoQ-TPP compounds

To synthesize CoQ_4_-TPP compounds, CoQ_4_ (100 mg, 2.26 μmol) was added to isopropanol/methanol (10 ml). CoQ_4_ for synthesis is obtained from WuXi Chemicals. NaBH_4_ (Sigma, 480886) was added to CoQ until color change from orange to colorless was observed, indicating reduction. Reaction quenched with 1M hydrochloric acid (20 ml), diluted with dichloromethane (15 ml), and washed with 1M hydrochloric acid (50 ml) then brine (50 ml). Organic layer was sealed under argon. 4-dimethylaminopyridine (25 mg, 2.0 μmol; Sigma, 107700), N-(3-dimethylaminopropyl)-N’ethylcarbodiimide hydrochloride (45 mg, 2.0 μmol; Sigma, 03450), and 4-carboxybutyl- (Sigma, 157945); 6-carboxyhexyl (WuXi Chemicals) or 8-carboxyoctyl (Toronto Research Chemicals, C178905) triphenylphosphonium bromide (100 mg, 2.0 μmol) were dissolved in dichloromethane and added dropwise to reduced CoQ. Reaction was stirred at room temperature for 72 h, and product formation was confirmed by TLC. Solvent was removed under reduced pressure, and column chromatography on silica gel with 2:1 ethyl acetate:methanol gave products as yellow gel. Remaining silica gel was filtered with acetonitrile elution. The yield calculated at 5 to 10% for monoacylated products.

CoQ_4_-TPP_4_: ^1^H NMR 7.87 (t, 6H), 7.77 (t, 3H), 7.68 (t, 6H), 5.09 (m, 4H), 4.00 (m, 2H), 3.87 (m, 2H), 3.67 (s, 2H), 3.65 (s, 2H), 3.32 (d, 1H), 3.09 (d, 1H), 2.11 (d, 3H), 2.05 (d, 5H, 1.97 (d, 5H), 1.68 (s, 9H), 1.60 (m, 12H), 1.23 (s, 6H), 0.89 (m, 3H). ^13^C NMR 134.89, 133.85, 130.49, 124.42, 118.92, 118.24, 96.15, 60.83, 39.75, 32.94, 29.72, 26.79, 25.72, 25.42, 21.88, 20.39, 17.72, 16.03, 12.12. HRMS (ESI-MS, m/z) calculated 801.46, found 801.465.

CoQ_4_-TPP_6_: ^1^H NMR 7.89 (t, 6H), 7.78 (t, 3H), 7.70 (t, 6H), 5.09 (m, 4H), 3.97 (m, 2H), 3.88 (m, 2H), 3.76 (s, 2H), 3.33 (d, 1H), 3.14 (d, 1H), 2.56 (M, 2H), 2.12 (s, 2H), 2.05 (d, 5H, 1.97 (d, 6H), 1.76 (s, 15H), 1.68 (m, 9H), 1.60 (s, 9H), 1.46 (m, 3H), 0.93 (m, 2H). ^13^C NMR 172.11, 144.87, 137.64, 134.89, 133.75, 130.4, 124.43, 118.99, 118.3, 96.15, 60.98, 39.75, 33.72, 29.81, 28.53, 26.80, 25.73, 24.5, 22.45, 17.72, 16.02, 12.12. HRMS (ESI-MS, m/z) calculated 829.50, found 829.495.

CoQ_4_-TPP_8_: ^1^H NMR 7.86 (t, 6H), 7.79 (t, 3H), 7.71 (t, 6H), 5.10 (m, 4H), 4.0 (m, 2H), 3.9 (m, 3H), 3.79 (m, 2H), 3.7 (m, 1H), 3.34 (d, 1H), 3.2 (d, 1H), 2.55 (m, 1H), 2.48 (m, 1H), 2.05 (m, 6H), 2.0 (m, 6H), 1.81 (m, 18H), 1.68 (s, 6H), 1.60 (m, 12H), 1.26 (m, 9H), 0.89 (m, 2H). ^13^C NMR 135.99, 134.87, 133.74, 130.50, 130.40, 124.42, 119.02, 118.34, 96.14, 39.75, 29.73, 28.83, 27.36, 26.79, 25.73, 24.99, 24.15, 22.72, 17.72, 16.05. HRMS (ESI-MS, m/z) calculated 857.53, found 857.526.

### High performance liquid chromatography–electrochemical detection measurement of CoQ levels

Samples were injected into HPLC (Ultimate 3000, Thermo Scientific) with an electrochemical detector (ECD-3000RS). The first electrode (6020RS) was set to +600 mV and placed before the column (Thermo Scientific, Betasil C18, 100 × 2.1 mm, 3 μM particle) to oxidize all quinones. The second electrode (6011RS) was set to −600 mV to reduce all quinones exiting the column, and the third electrode was set at +600 mV to measure redox active species. Peaks were quantified with Chromeleon 7.2.10 software. To measure CoQ_4_ levels, extracted lipids were resuspended in methanol, and the mobile phase was 95% methanol 5% 1M ammonium acetate pH 4.4 in water. To measure CoQ_10_ levels, extracted lipids were resuspended in isopropanol, and the mobile phase was 78% methanol, 20% isopropanol, and 2% 1M ammonium acetate pH 4.4 in water.

### Representative esterase hydrolysis

CoQ-TPP compounds were incubated at 200 μM at 37 °C for 24 h in KCl buffer (120 mM KCl, 10 mM Hepes, 1 mM EGTA, pH = 7.2) containing either 1 mg/ml porcine liver esterase (Sigma, E2884) or a buffer control along with CoQ_2_ (Sigma, C8081) as an internal standard. To extract CoQ_4_, cold LC-MS grade methanol was added, and sample was incubated at −80 °C for 15 min. Sample was vortexed for 5 min at 4 °C in a disruptor genie set to max (3000 rpm) and centrifuged at 16,000*g* for 5 min. Supernatant was collected and dried under nitrogen flow, and CoQ species were measured by high performance liquid chromatography–electrochemical detection (HPLC-ECD). To extract CoQ_10_, 400 μl petroleum ether was added to reaction. Samples were vortexed for 10 min at 4 °C in a disruptor genie set to max (3000 rpm), then centrifuged at 1000*g* for 3 min. Petroleum ether layer was collected, and 400 μl fresh petroleum ether added to reaction mixture. Samples were vortexed for 3 min at 4 °C in a disruptor genie set to max (3000 rpm), then centrifuged at 1000*g* for 3 min. Petroleum ether layer was collected and combined with prior petroleum ether, dried under nitrogen flow, and CoQ species measured by HPLC-ECD.

### Extraction of CoQ species

Wildtype HepG2 cells wells were seeded at 3,000,000 cells/well in a 6-well plate and allowed to adhere overnight. Medium was exchanged to DMEM containing either vehicle (isopropanol), CoQ_4_ (Sigma, C2470), or CoQ_4_-TPP. After 24 h, cells were trypsinized and pelleted (600*g*, 5 min). Pellet was washed with dPBS) + 10% isopropanol followed by dBPS + 30% isopropanol. Pellet was flash frozen in 500 μl cold methanol with 2 μM of CoQ_6_ (Avanti, 900150O) as an internal standard. Cells were lysed by vortexing for 10 min at 4 °C in a disruptor genie set to max (3000 rpm) speed. Sample was spun 5 min 16,000*g*, and the supernatant was collected. Five hundred microliter of cold methanol was added, and sample was vortexed for 3 min and then spun as above. Supernatant was again collected and combined with supernatant from previous step and dried under nitrogen gas.

### Mitochondria subfractionation and CoQ extraction

HepG2 cells were seeded in 15 cm plates and allowed to adhere overnight. Media were exchanged for DMEM with 10 μM either CoQ_4_-TPP or CoQ_4_ (Sigma, C2470). After 3 h, mitochondrial fractions were isolated from cells through differential centrifugation as described previously (Frezza, 2007). Briefly, cells and media were collected and washed with dPBS then dPBS + 10% isopropanol. Cells were resuspended in isolation buffer (10 mM Tris-Mops, pH 7.4, 1 mM EGTA/Tris, 200 mM sucrose) and homogenized with 50 strokes of a glass-Teflon potter. Whole cell and nuclei were pelleted (10 min × 600*g*, 4 °C), supernatant collected and centrifuged to produce mitochondrial fraction (10 min × 7000*g*, 4 °C). Supernatant of hard spin collected as cytoplasmic fraction. Protein content in fractions was quantified using Pierce BCA Protein Assay Kit. (Thermo, 23225). Fifty microgram protein from each fraction saved for later analysis by Western blot.

To extract CoQ_4_, the mitochondrial fraction was resuspended in an equivalent amount of isolation buffer to cytoplasmic fraction. Four hundred microliters of chloroform with CoQ_6_ (Avanti, 900150O) as an internal standard was added, and the sample was vortexed for 10 min at 4 °C in a disruptor genie set to max (3000 rpm) speed. Chloroform was collected, and process is repeated. Lipids were dried and measured with HPLC-ECD.

To assess fraction purity, the 50 μg protein collected was methanol precipitated. Sample was solubilized in radioimmunoprecipitation assay buffer, and 1 ml methanol was added. Sample was pelleted (20 min, 16,000*g*), and supernatant was discarded. 1 ml 90:10 methanol:water (v/v) was added, sample was pelleted (20 min, 16,000*g*), and supernatant was discarded. Precipitated protein was resuspended in sample buffer (Invitrogen, NP0007) to a final concentration of 1 mg/ml. Ten microgram of protein from each sample was separated on a NuPAGE 10% Bis-Tris gel (Thermo, NP0303BOX) with a protein standard (Licor, 928-60000). Resolved proteins were then transferred to PVDF membrane (Fisher, IPFL00010), probed with primary antibodies (Abcam, 154856; Cell Signaling, 9644S) followed by HRP-conjugated secondary antibody (Cell Signaling Technologies, 7074S, 7076S) and ECL substrate (Thermo, 34579, 34094). Blots were imaged and analyzed using Azure5 Imaging System (version 1.9.0.0406).

### CoQ-TPP toxicity cell counts

Wildtype HepG2 cells seeded at 150,000 cells/well in 6-well plate and allowed to adhere overnight. Media were exchanged for DMEM containing CoQ-TPP additives and incubated for 24 h. Cells were washed with dPBS, collected, and counted using a Muse Cell Analyzer and the Muse Count and Viability Assay (Luminex).

## Data availability

All data contained within the manuscript.

## Supporting information

This article contains supporting information.

## Conflict of interest

The authors declare that they have no conflicts of interest with the contents of this article.
