# Supplementary material for: Coenzyme Q4 is a functional substitute for coenzyme Q10 and can be targeted to the mitochondria
Source: J Biol Chem. 2024 Apr 6;300(5):107269. doi: 10.1016/j.jbc.2024.107269 (PMC11087978; doi:10.1016/j.jbc.2024.107269)
Supplement: Supporting Figures [file mmc1.docx]

**SUPPORTING INFORMATION**


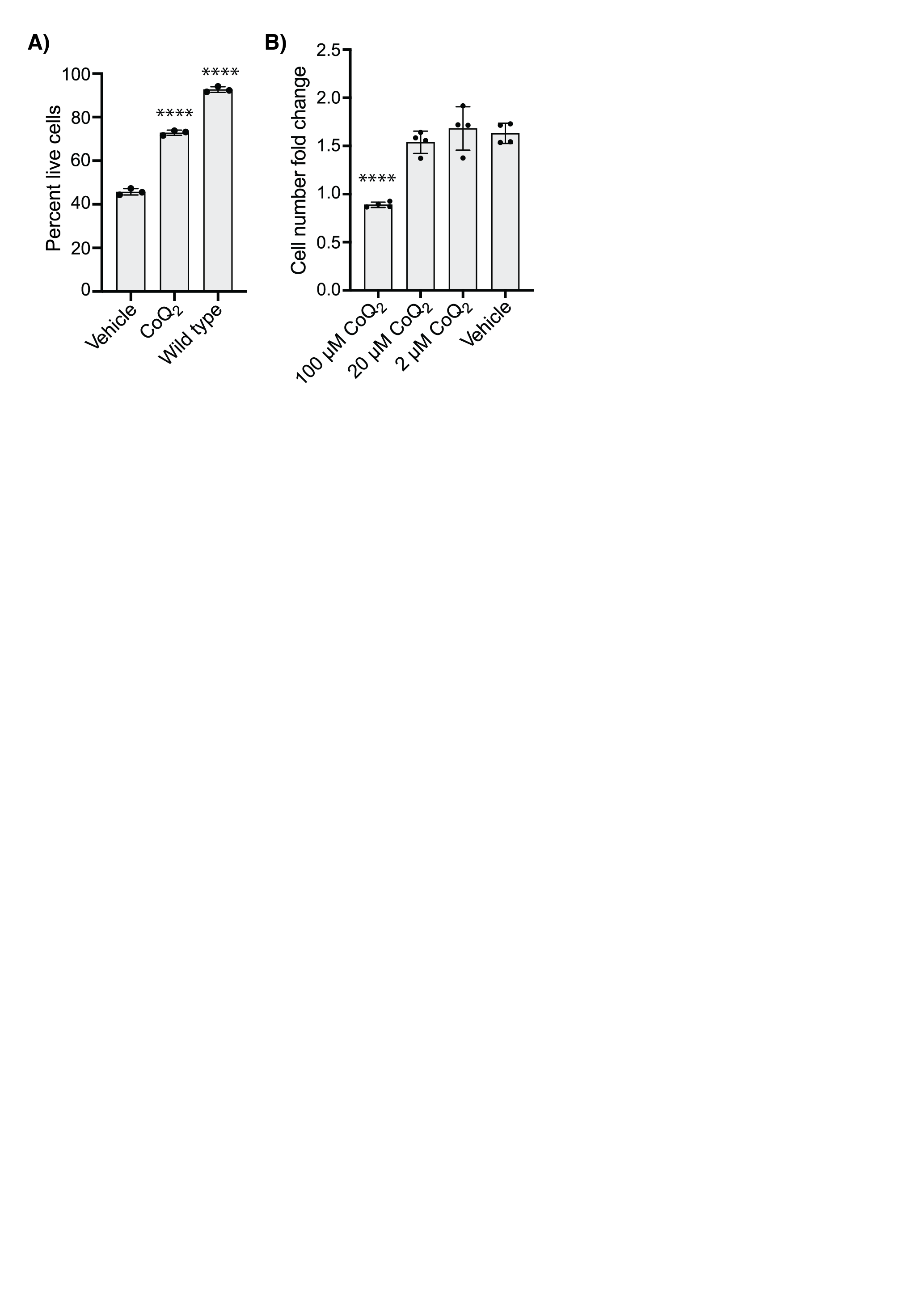


Supporting Information Figure S1. CoQ_2_ can support OxPhos but has inherent toxicity.

**a.** Percent non-apoptotic *COQ2^-/-^* and wild-type HepG2 cells after 48 hours of incubation in galactose media supplemented with 20 µM CoQ_2_ or vehicle (isopropanol). Cells were pre-treated for 24 hours with CoQ_2_ to ensure sufficient uptake. Non-apoptotic cells defined as not staining for AAD-7 or annexin-V. *n* = three independent technical replicates, error bars indicate standard deviation.

**b.** Cell number fold change in wild-type HepG2 cells after 48 hours of incubation with indicated concentrations of CoQ_2_. *n* = four independent technical replicates, error bars indicate standard deviation.

***** p < 0.0001*

**
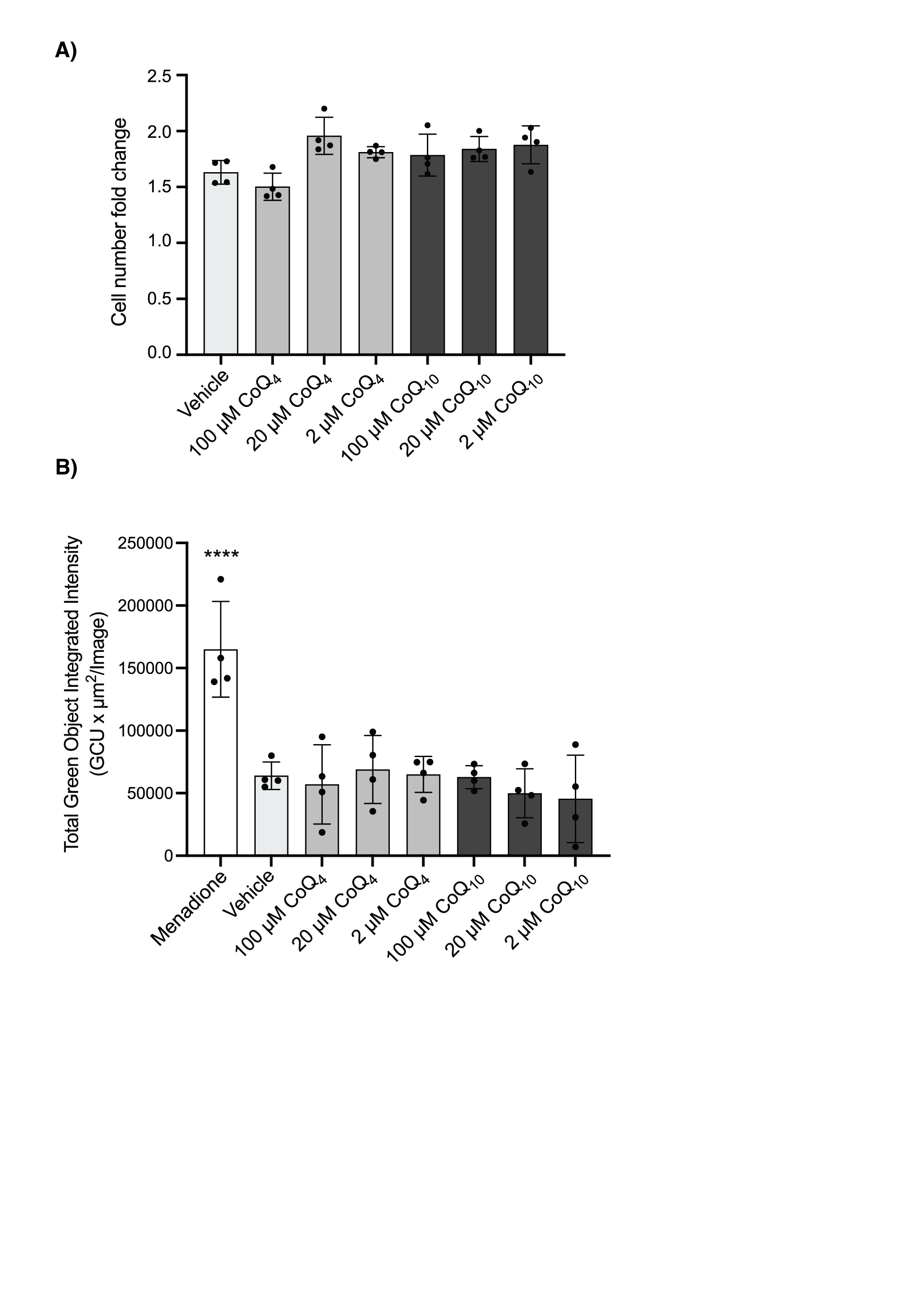
**

Supporting Information Figure S2. CoQ_4_ is not toxic at operative concentrations.

**a.** Cell number fold change in wild-type HepG2 cells after 48 hours of incubation with indicated concentrations of CoQ_4_ or CoQ_10_.

**b.** ROS production as visualized by green fluorescence from DCFDA in wild-type HepG2 cells after 24 hours of treatment with a positive control (25µM menadione) or indicated CoQ additives.

*n* = four independent technical replicates, error bars indicate standard deviation.

***** p < 0.0001*


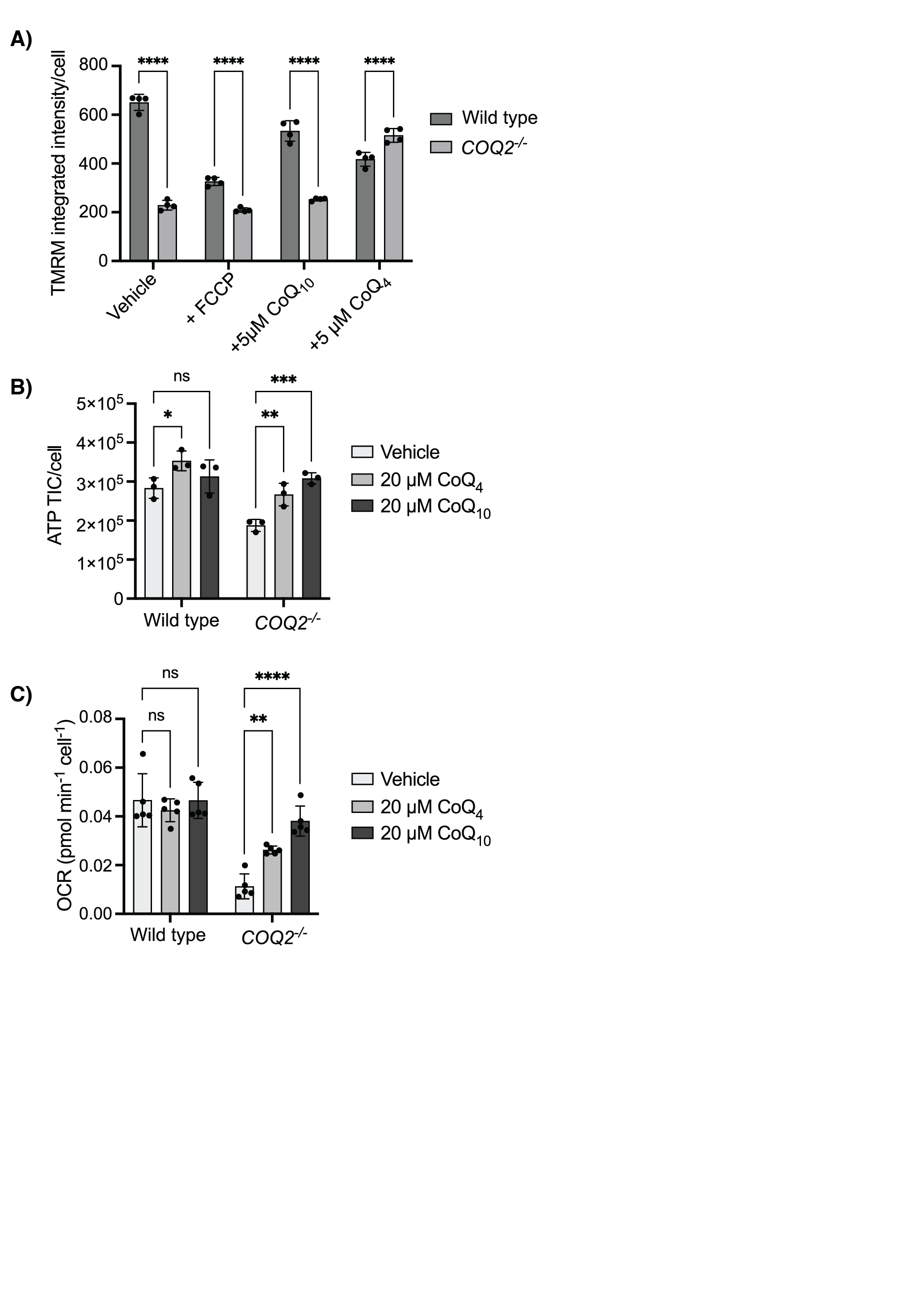


**Supporting Information Figure S3. CoQ_4_ alleviates multiple mitochondrial disruptions caused by CoQ deficiency.**

**a.** Mitochondrial membrane potential in wild-type or *COQ2^-/-^* HepG2 cells as measured by TMRM fluorescence. Cells were incubated with 5 µM CoQ_4_, CoQ_10_, or vehicle in galactose media. For FCCP measurements, cells were incubated with 6 µM FCCP prior to measurement. *n* = four independent technical replicates, error bars indicate standard deviation.

**b.** Total ion counts of ATP normalized to cell count in HepG2 wild-type and *COQ2^-/-^* cells after treatment with 20 µM CoQ_4_, CoQ_10_, or vehicle in galactose media. *n* = three independent technical replicates, error bars indicate standard deviation.

**c.** Oxygen consumption rate as determined by Seahorse in HepG2 wild-type and *COQ2^-/-^* cells after treatment with 20 µM CoQ_4_, CoQ_10_, or vehicle in galactose media. *n* = five independent technical replicates, error bars indicate standard deviation.

*ns: not significant, * p < 0.05, ** p < 0.01, *** p < 0.001,**** p < 0.0001*

*
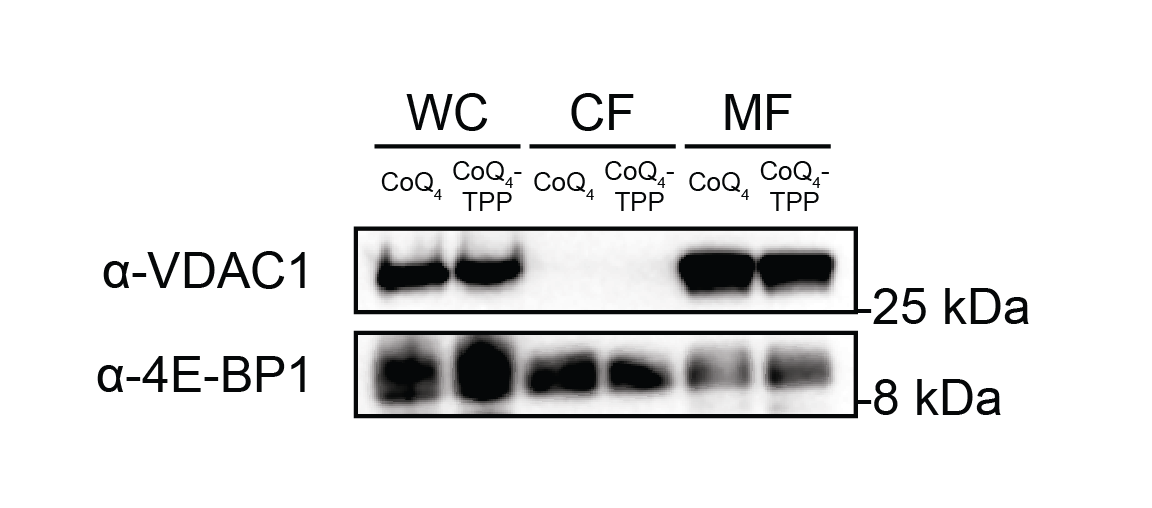
*

**Supporting Information Figure S4. Crude mitochondria isolation blot.**

Enrichment of α-VDAC1 (mitochondrial marker) or α-4E-BP1 (cytoplasmic marker) in samples after mitochondrial preparation. WC – whole cell, CF – cytoplasmic fraction, MF – mitochondrial fraction.


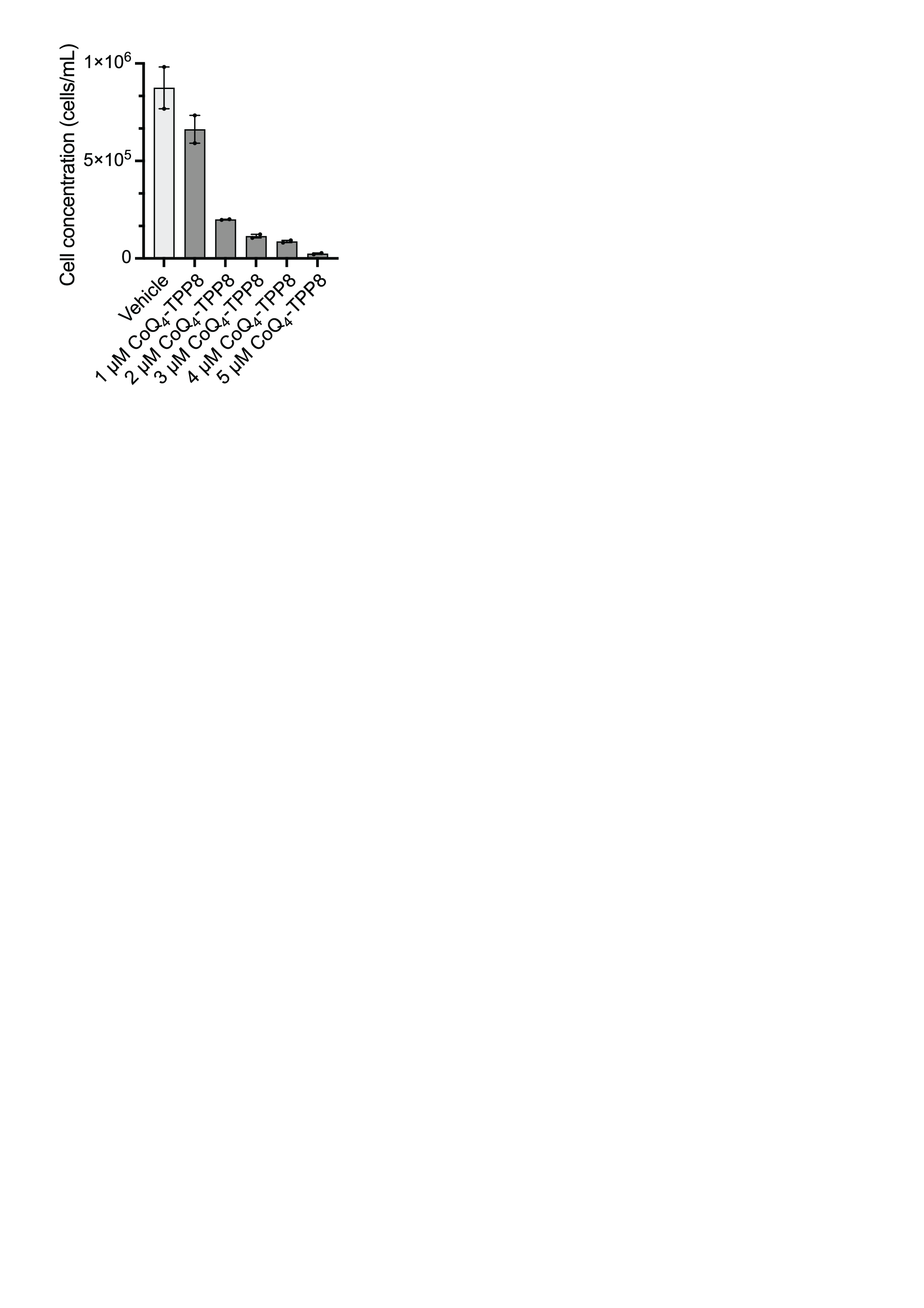


**Supporting Information Figure S5. CoQ_4_-TPP compounds cause cell death**.

Cell concentration after 24 hours of incubation with various concentrations of CoQ_4_-TPP8.

*n* = two independent technical replicates, error bars indicate standard deviation.
